# 5-Chloro-1*H*-indole-3-carb­oxy­lic acid

**DOI:** 10.1107/S1600536811053384

**Published:** 2011-12-17

**Authors:** Xue-Lian Han, Yang-Hui Luo

**Affiliations:** a College of Chemistry and Chemical Engineering, Southeast University, Nanjing 210096, People’s Republic of China

## Abstract

In the title compound, C_9_H_6_ClNO_2_, the carboxyl group is twisted from the indole ring system by 9.00 (8)°. In the crystal, inversion dimers linked by pairs of O—H⋯O hydrogen bonds generate *R*_2_^2^(8) loops and N—H⋯O hydrogen bonds link the dimers into (001) sheets. Aromatic π–π stacking inter­actions [centroid–centroid distance = 3.7185 (12) A °] are also observed.

## Related literature

For background to indole derivatives as pharmaceuticals, see: Kunzer & Wendt (2011 ▶); Woodward & Bartel (2005 ▶).
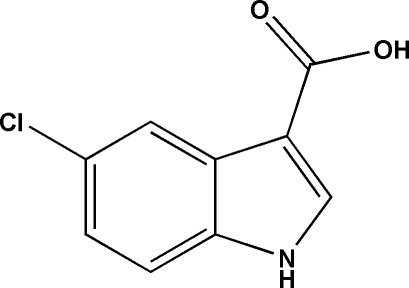


## Experimental

### 

#### Crystal data

C_9_H_6_ClNO_2_
*M* *_r_* = 195.60
Orthorhombic, 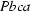

*a* = 7.2934 (15) Å
*b* = 13.065 (3) Å
*c* = 17.902 (4) Å
*V* = 1705.9 (6) Å^3^
*Z* = 8
Mo *K*α radiation
μ = 0.41 mm^−1^
*T* = 293 K
0.30 × 0.20 × 0.15 mm

#### Data collection

Rigaku SCXmini CCD diffractometer
Absorption correction: multi-scan (*CrystalClear*; Rigaku, 2005 ▶) *T*_min_ = 0.907, *T*_max_ = 0.941
16050 measured reflections
1919 independent reflections
1590 reflections with *I* > 2σ(*I*)
*R*_int_ = 0.050

#### Refinement

*R*[*F* ^2^ > 2σ(*F* ^2^)] = 0.037
*wR*(*F* ^2^) = 0.104
*S* = 1.04
1919 reflections
123 parameters
H atoms treated by a mixture of independent and constrained refinement
Δρ_max_ = 0.17 e Å^−3^
Δρ_min_ = −0.23 e Å^−3^

### 

Data collection: *CrystalClear* (Rigaku, 2005 ▶); cell refinement: *CrystalClear*; data reduction: *CrystalClear*; program(s) used to solve structure: *SHELXS97* (Sheldrick, 2008 ▶); program(s) used to refine structure: *SHELXL97* (Sheldrick, 2008 ▶); molecular graphics: *DIAMOND* (Brandenburg & Putz, 2005 ▶); software used to prepare material for publication: *SHELXL97*.

## Supplementary Material

Crystal structure: contains datablock(s) I, global. DOI: 10.1107/S1600536811053384/hb6559sup1.cif

Structure factors: contains datablock(s) I. DOI: 10.1107/S1600536811053384/hb6559Isup2.hkl

Supplementary material file. DOI: 10.1107/S1600536811053384/hb6559Isup3.cml

Additional supplementary materials:  crystallographic information; 3D view; checkCIF report

## Figures and Tables

**Table 1:** Hydrogen-bond geometry (Å, °)

*D*—H⋯*A*	*D*—H	H⋯*A*	*D*⋯*A*	*D*—H⋯*A*
O1—H1⋯O2^i^	0.82	1.86	2.6558 (17)	164
N1—H1*B*⋯O2^ii^	0.86 (2)	2.26 (2)	3.005 (2)	144.3 (19)

Symmetry codes: (i) 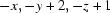; (ii) 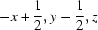.

**Fig. 1.**
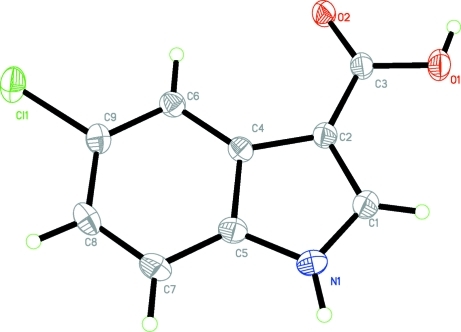
The molecular structure of the title compound with displacement ellipsoids drawn at the 30% probability level.

**Fig. 2.**
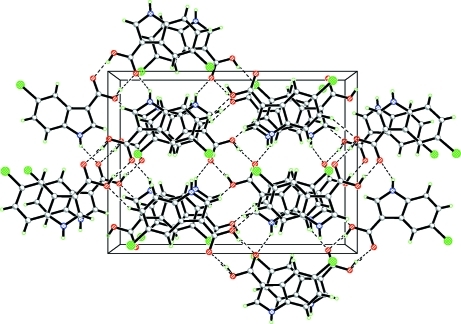
A packing view down the a axis showing hydrogen bonds as dashed lines.

## supplementary crystallographic information

### Comment

Indole-3-carboxylic acid and its derivatives are important chemical materials, because they are excellent auxins for plants and drug intermediates for many pharmaceutical products (Woodward, *et al.*,2005, Kunzer, *et al.*,2011). As part of our interest in these materials, we report here the crystal structure of the title compound.

The molecular structure of the title compound is shown in Fig. 1. All the non-H atoms are approximately coplanar: the carboxy O atoms deviating by 0.124 and -0.223 Å from the indole plane.

In the crystal, O—H···O hydrogen bonds linked the molecules into dimers and the dimers packed *via* N—H···O hydrogen bonds and π–π interactions [centroid–centroid distance = 3.7185 (12) A °] (Fig. 2).

### Experimental

The title compound was purchased from ChemFuture PharmaTech, Ltd (Nanjing, Jiangsu).  Colourless blocks were obstained by slow evaporation of a methanol solution.

### Refinement

All H atoms attached to C, N and O atoms were fixed geometrically and treated as riding with C—H = 0.93 Å (CH), O—H = 0.82 Å and N—H = 0.86 Å with *U*_iso_(H) = 1.2*U*_eq_(CH), *U*_iso_(H) = 1.35*U*_eq_(N) and *U*_iso_(H) = 1.5*U*_eq_(O).

### Crystal data

**Table:**

C_9_H_6_ClNO_2_	*F*(000) = 800
*M**_r_* = 195.60	*D*_x_ = 1.523 Mg m^−^^3^
Orthorhombic, *P**b**c**a*	Mo *K*α radiation, λ = 0.71073 Å
Hall symbol: -P 2ac 2ab	Cell parameters from 1919 reflections
*a* = 7.2934 (15) Å	θ = 3.1–27.4°
*b* = 13.065 (3) Å	µ = 0.41 mm^−^^1^
*c* = 17.902 (4) Å	*T* = 293 K
*V* = 1705.9 (6) Å^3^	Block, colourless
*Z* = 8	0.30 × 0.20 × 0.15 mm

### Data collection

**Table:**

Rigaku SCXmini CCD diffractometer	1919 independent reflections
Radiation source: fine-focus sealed tube	1590 reflections with *I* > 2σ(*I*)
graphite	*R*_int_ = 0.050
Detector resolution: 13.6612 pixels mm^-1^	θ_max_ = 27.4°, θ_min_ = 3.1°
CCD_Profile_fitting scans	*h* = −9→9
Absorption correction: multi-scan (*CrystalClear*; Rigaku, 2005)	*k* = −16→16
*T*_min_ = 0.907, *T*_max_ = 0.941	*l* = −23→23
16050 measured reflections	

### Refinement

**Table:**

Refinement on *F*^2^	Primary atom site location: structure-invariant direct methods
Least-squares matrix: full	Secondary atom site location: difference Fourier map
*R*[*F*^2^ > 2σ(*F*^2^)] = 0.037	Hydrogen site location: inferred from neighbouring sites
*wR*(*F*^2^) = 0.104	H atoms treated by a mixture of independent and constrained refinement
*S* = 1.04	*w* = 1/[σ^2^(*F*_o_^2^) + (0.052*P*)^2^ + 0.5573*P*] where *P* = (*F*_o_^2^ + 2*F*_c_^2^)/3
1919 reflections	(Δ/σ)_max_ < 0.001
123 parameters	Δρ_max_ = 0.17 e Å^−^^3^
0 restraints	Δρ_min_ = −0.23 e Å^−^^3^

### Special details

**Table:**

Geometry. All e.s.d.'s (except the e.s.d. in the dihedral angle between two l.s. planes) are estimated using the full covariance matrix. The cell e.s.d.'s are taken into account individually in the estimation of e.s.d.'s in distances, angles and torsion angles; correlations between e.s.d.'s in cell parameters are only used when they are defined by crystal symmetry. An approximate (isotropic) treatment of cell e.s.d.'s is used for estimating e.s.d.'s involving l.s. planes.
Refinement. Refinement of *F*^2^ against ALL reflections. The weighted *R*-factor *wR* and goodness of fit *S* are based on *F*^2^, conventional *R*-factors *R* are based on *F*, with *F* set to zero for negative *F*^2^. The threshold expression of *F*^2^ > σ(*F*^2^) is used only for calculating *R*-factors(gt) *etc*. and is not relevant to the choice of reflections for refinement. *R*-factors based on *F*^2^ are statistically about twice as large as those based on *F*, and *R*- factors based on ALL data will be even larger.

### Fractional atomic coordinates and isotropic or equivalent isotropic displacement parameters (Å^2^)

**Table:**

	*x*	*y*	*z*	*U*_iso_*/*U*_eq_	
Cl1	0.07292 (7)	0.95139 (4)	0.09595 (2)	0.05145 (18)	
O2	0.05698 (16)	0.98847 (9)	0.40623 (6)	0.0402 (3)	
C3	0.0835 (2)	0.89840 (12)	0.42814 (9)	0.0343 (3)	
N1	0.2225 (2)	0.65957 (11)	0.33911 (8)	0.0428 (4)	
C4	0.1415 (2)	0.81729 (11)	0.29670 (8)	0.0301 (3)	
C6	0.1018 (2)	0.89102 (11)	0.24148 (8)	0.0329 (3)	
H6	0.0636	0.9566	0.2542	0.040*	
O1	0.0656 (2)	0.87130 (10)	0.49869 (6)	0.0568 (4)	
H1	0.0422	0.9220	0.5240	0.085*	
C5	0.1970 (2)	0.71749 (12)	0.27449 (9)	0.0350 (4)	
C2	0.1353 (2)	0.81582 (11)	0.37768 (9)	0.0322 (3)	
C9	0.1218 (2)	0.86192 (13)	0.16732 (9)	0.0365 (4)	
C7	0.2150 (2)	0.68926 (13)	0.19938 (10)	0.0429 (4)	
H7	0.2508	0.6235	0.1860	0.052*	
C1	0.1853 (2)	0.71802 (13)	0.39985 (9)	0.0403 (4)	
H1A	0.1924	0.6958	0.4491	0.048*	
C8	0.1775 (2)	0.76284 (14)	0.14570 (9)	0.0434 (4)	
H8	0.1890	0.7468	0.0953	0.052*	
H1B	0.265 (3)	0.5983 (18)	0.3404 (11)	0.058 (6)*	

### Atomic displacement parameters (Å^2^)

**Table:**

	*U*^11^	*U*^22^	*U*^33^	*U*^12^	*U*^13^	*U*^23^
Cl1	0.0648 (3)	0.0559 (3)	0.0337 (2)	−0.0029 (2)	−0.00505 (19)	0.00701 (18)
O2	0.0590 (8)	0.0296 (6)	0.0320 (6)	0.0048 (5)	0.0087 (5)	0.0028 (4)
C3	0.0418 (9)	0.0338 (8)	0.0274 (7)	0.0000 (6)	0.0024 (6)	0.0008 (6)
N1	0.0569 (9)	0.0271 (7)	0.0443 (8)	0.0067 (6)	0.0023 (7)	0.0004 (6)
C4	0.0302 (7)	0.0293 (7)	0.0307 (8)	−0.0027 (6)	0.0013 (6)	−0.0031 (6)
C6	0.0350 (8)	0.0308 (7)	0.0331 (8)	−0.0013 (6)	0.0009 (6)	−0.0020 (6)
O1	0.1032 (12)	0.0388 (7)	0.0285 (6)	0.0165 (7)	0.0126 (7)	0.0045 (5)
C5	0.0353 (8)	0.0309 (8)	0.0387 (8)	−0.0004 (6)	0.0011 (6)	−0.0030 (6)
C2	0.0370 (8)	0.0302 (8)	0.0295 (8)	0.0001 (6)	0.0013 (6)	0.0011 (6)
C9	0.0369 (8)	0.0419 (9)	0.0306 (8)	−0.0046 (7)	−0.0015 (6)	−0.0005 (7)
C7	0.0498 (10)	0.0358 (9)	0.0432 (9)	0.0022 (7)	0.0038 (8)	−0.0122 (7)
C1	0.0526 (10)	0.0346 (8)	0.0338 (8)	0.0037 (7)	0.0013 (7)	0.0027 (6)
C8	0.0493 (10)	0.0496 (10)	0.0314 (8)	−0.0036 (8)	0.0023 (7)	−0.0119 (7)

### Geometric parameters (Å, °)

**Table:**

Cl1—C9	1.7681 (17)	C6—C9	1.389 (2)
O2—C3	1.256 (2)	C6—H6	0.9300
C3—O1	1.3181 (19)	O1—H1	0.8200
C3—C2	1.457 (2)	C5—C7	1.400 (2)
N1—C1	1.356 (2)	C2—C1	1.387 (2)
N1—C5	1.395 (2)	C9—C8	1.411 (2)
N1—H1B	0.86 (2)	C7—C8	1.387 (3)
C4—C6	1.410 (2)	C7—H7	0.9300
C4—C5	1.422 (2)	C1—H1A	0.9300
C4—C2	1.451 (2)	C8—H8	0.9300
			
O2—C3—O1	122.39 (14)	C1—C2—C4	106.86 (13)
O2—C3—C2	122.71 (14)	C1—C2—C3	124.96 (14)
O1—C3—C2	114.89 (14)	C4—C2—C3	128.16 (14)
C1—N1—C5	109.45 (14)	C6—C9—C8	122.94 (15)
C1—N1—H1B	125.1 (14)	C6—C9—Cl1	119.25 (13)
C5—N1—H1B	125.2 (14)	C8—C9—Cl1	117.81 (12)
C6—C4—C5	119.26 (14)	C8—C7—C5	117.67 (15)
C6—C4—C2	134.69 (14)	C8—C7—H7	121.2
C5—C4—C2	106.02 (13)	C5—C7—H7	121.2
C9—C6—C4	117.48 (14)	N1—C1—C2	109.99 (15)
C9—C6—H6	121.3	N1—C1—H1A	125.0
C4—C6—H6	121.3	C2—C1—H1A	125.0
C3—O1—H1	109.5	C7—C8—C9	120.19 (15)
N1—C5—C7	129.87 (15)	C7—C8—H8	119.9
N1—C5—C4	107.67 (14)	C9—C8—H8	119.9
C7—C5—C4	122.45 (15)		

### Hydrogen-bond geometry (Å, °)

**Table:**

*D*—H···*A*	*D*—H	H···*A*	*D*···*A*	*D*—H···*A*
O1—H1···O2^i^	0.82	1.86	2.6558 (17)	164
N1—H1B···O2^ii^	0.86 (2)	2.26 (2)	3.005 (2)	144.3 (19)

Symmetry codes: (i) −*x*, −*y*+2, −*z*+1; (ii) −*x*+1/2, *y*−1/2, *z*.
